# Iatrogenic Creutzfeldt-Jakob Disease, Final Assessment

**DOI:** 10.3201/eid1806.120116

**Published:** 2012-06

**Authors:** Paul Brown, Jean-Philippe Brandel, Takeshi Sato, Yosikazu Nakamura, Jan MacKenzie, Robert G. Will, Anna Ladogana, Maurizio Pocchiari, Ellen W. Leschek, Lawrence B. Schonberger

**Affiliations:** Centre à l’Energie Atomique, Fontenay-aux-Roses, France (P. Brown);; Institut National de la Santé et de la Recherche Médicale, Paris, France (J.-P. Brandel);; Nanohana Clinic, Tokyo, Japan (T. Sato);; Jichi Medical University, Yakushiji, Japan (Y. Nakamura);; Western General Hospital, Edinburgh, Scotland, UK (J. MacKenzie, R.G. Will);; Istituto Superiore de Sanità, Rome, Italy (A. Ladogana, M. Pocchiari);; National Institutes of Health, Bethesda, Maryland, USA (E.W. Leschek);; Centers for Disease Control and Prevention, Atlanta, Georgia, USA (L.B. Schonberger)

**Keywords:** Creutzfeldt-Jakob disease, dura mater, human growth hormone, iatrogenic disease, *PRNP* codon 129, variant Creutzfeldt-Jakob disease, prions and related diseases

## Abstract

The book on iatrogenic Creutzfeldt-Jakob disease (CJD) in humans is almost closed. This form of CJD transmission via medical misadventures was first detected in 1974. Today, only occasional CJD cases with exceptionally long incubation periods still appear. The main sources of the largest outbreaks were tissues from human cadavers with unsuspected CJD that were used for dura mater grafts and growth hormone extracts. A few additional cases resulted from neurosurgical instrument contamination, corneal grafts, gonadotrophic hormone, and secondary infections from blood transfusions. Although the final solution to the problem of iatrogenic CJD is still not available (a laboratory test to identify potential donors who harbor the infectious agent), certain other measures have worked well: applying special sterilization of penetrating surgical instruments, reducing the infectious potential of donor blood and tissue, and excluding donors known to have higher than normal risk for CJD.

## Medscape CME Activity

Medscape, LLC is pleased to provide online continuing medical education (CME) for this journal article, allowing clinicians the opportunity to earn CME credit.

This activity has been planned and implemented in accordance with the Essential Areas and policies of the Accreditation Council for Continuing Medical Education through the joint sponsorship of Medscape, LLC and Emerging Infectious Diseases. Medscape, LLC is accredited by the ACCME to provide continuing medical education for physicians.

Medscape, LLC designates this Journal-based CME activity for a maximum of 1 *AMA PRA Category 1 Credit(s)*^TM^. Physicians should claim only the credit commensurate with the extent of their participation in the activity.

All other clinicians completing this activity will be issued a certificate of participation. To participate in this journal CME activity: (1) review the learning objectives and author disclosures; (2) study the education content; (3) take the post-test with a 70% minimum passing score and complete the evaluation at www.medscape.org/journal/eid; (4) view/print certificate.

**Release date: May 16, 2012; Expiration date: May 16, 2013**

## Learning Objectives

Upon completion of this activity, participants will be able to:

- Distinguish the principal sources of iatrogenic CJD
- Identify countries with the highest rates of documented CJD
- Analyze the clinical presentation of iatrogenic CJD
- Assess new threats which might promote higher rates of CJD.

## CME Editor

**P. Lynne Stockton,** Technical Writer/Editor, *Emerging Infectious Diseases. Disclosure: P. Lynne Stockton has disclosed no relevant financial relationships*.

## CME Author

**Charles P. Vega, MD,** Health Sciences Clinical Professor; Residency Director, Department of Family Medicine, University of California, Irvine. *Disclosure: Charles P. Vega, MD, has disclosed no relevant financial relationships.*

## Authors

*Disclosures:* ***Paul Brown; Jean-Philippe Brandel; Takeshi Sato, MD; Yosikazu Nakamura, MD, MPH, FFPH; Jan MacKenzie; Anna Ladogana; Ellen W. Leschek, MD;*** *and* ***Lawrence B. Schonberger, MD, MPH,*** *have disclosed no relevant financial relationships.* ***Robert G. Will, FRCP,*** *has disclosed the following relevant financial relationships: served as an advisor or consultant for LFB, Farring.* ***Maurizio Pocchiari, MD,*** *has disclosed the following relevant financial relationships: served as an advisor or consultant for LFB, Farring.*
