# Supplementary material for: Iatrogenic Creutzfeldt-Jakob Disease, Final Assessment
Source: Emerg Infect Dis. 2012 Jun;18(6):901–7. doi: 10.3201/eid1806.120116 (PMC3358170; doi:10.3201/eid1806.120116)
Supplement: Related texts

# Review article: Iatrogenic Creutzfeldt-Jakob Disease, Final Assessment

**DOI:** 10.3201/eid1806.120116

**Authors:** Paul Brown, Jean-Philippe Brandel, Takeshi Sato, Yosikazu Nakamura, Jan MacKenzie, Robert G. Will, Anna Ladogana, Maurizio Pocchiari, Ellen W. Leschek, Lawrence B. Schonberger

**Affiliations:** Centre à l’Energie Atomique, Fontenay-aux-Roses, France (P. Brown);; Institut National de la Santé et de la Recherche Médicale, Paris, France (J.-P. Brandel);; Nanohana Clinic, Tokyo, Japan (T. Sato);; Jichi Medical University, Yakushiji, Japan (Y. Nakamura);; Western General Hospital, Edinburgh, Scotland, UK (J. MacKenzie, R.G. Will);; Istituto Superiore de Sanità, Rome, Italy (A. Ladogana, M. Pocchiari);; National Institutes of Health, Bethesda, Maryland, USA (E.W. Leschek);; Centers for Disease Control and Prevention, Atlanta, Georgia, USA (L.B. Schonberger)

## Abstract

The era of iatrogenic Creutzfeldt-Jakob disease (CJD) has nearly closed; only occasional cases with exceptionally long incubation periods are still appearing. The principal sources of these outbreaks are contaminated growth hormone (226 cases) and dura mater grafts (228 cases) derived from human cadavers with undiagnosed CJD infections; a small number of additional cases are caused by neurosurgical instrument contamination, corneal grafts, gonadotrophic hormone, and secondary infection with variant CJD transmitted by transfusion of blood products. No new sources of disease have been identified, and current practices, which combine improved recognition of potentially infected persons with new disinfection methods for fragile surgical instruments and biological products, should continue to minimize the risk for iatrogenic disease until a blood screening test for the detection of preclinical infection is validated for human use.

The first case of what would eventually become a major outbreak of iatrogenic Creutzfeldt-Jakob disease (CJD) was reported in 1974; the patient had received a corneal transplant from an infected cadaver (*1*). In the years that followed, other sources of infection were identified: stereotactic electroencephalogram electrodes, neurosurgical instruments, cadaveric dura mater and pituitary glands, and, most recently, secondary variant CJD (vCJD) blood products. The ensemble of iatrogenic cases, including a bibliography of primary references, was last reviewed in 2006 (*2*). Today, after nearly 40 years of surveillance, the chronology and essential characteristics of iatrogenic CJD have been finalized, and the purpose of this article is to present these data along with a few brief comments about factors that determined the risk for infection and how future risks might be foreseen and avoided.

By far the most common sources of iatrogenic disease were human cadavers from which pituitary hormones and dura mater grafts were obtained (Table 1; Figure); the other major variety of environmentally acquired disease is vCJD. The incidence curves of human growth hormone–associated and dura mater–associated CJD are almost superimposable; a broad peak occurred in the mid-to-late 1990s, just ahead of the sharper peak incidence of vCJD in the United Kingdom at the turn of the century. The incidence in other countries peaked a few years later, in 2004, as a result of the delayed appearance of bovine spongiform encephalopathy in those countries.

**Table 1:** Global distribution of cases of iatrogenic Creutzfeldt-Jakob disease*

Country	Source of infection and no. cases							
	Surgical procedure					Medical procedure		
	Dura mater grafts	Surgical instruments	EEG needles	Corneal transplants†		Growth hormone‡	Gonadotropin	Packed red blood cells§
Argentina	1							
Australia	5						4	
Austria	3					1		
Brazil						2		
Canada	4							
Croatia	1							
France	13	1				119		
Germany	10			1				
Ireland						1		
Italy	9							
Japan	142							
Netherlands	5					2		
New Zealand	2					6		
South Korea	2							
Qatar						1		
South Africa	1							
Spain	14							
Switzerland	3		2					
Thailand	1							
United Kingdom	8	3				65		3
United States	4			1		29		
Total	228	4	2	2		226	4	3

*EEG, electroencephalogram. †Additional possible single cases after corneal transplant or keratoplasty (not included in table) in Japan, United Kingdom, and United States. ‡Human growth hormone given in Brazil and New Zealand was prepared in the United States; that given in Qatar was prepared in France. Additional possible single cases with human growth hormone as source (not included in table) occurred in Sweden, Australia, and New Zealand. §An additional asymptomatic but infected red-cell recipient died of an unrelated illness; another asymptomatic infected hemophilia patient who had been exposed to potentially contaminated factor VIII also died of an unrelated illness (neither is included in the table).

**Figure:** Annual incidence of variant Creutzfeldt-Jakob disease (vCJD) caused by ingestion of meat products contaminated with bovine spongiform encephalopathy agent (A) and iatrogenic CJD caused by contaminated dura mater (B) and cadaveric human growth hormone (C), 1982–2011. White bars in panel A represent cases from outside the United Kingdom, which were delayed in parallel with the later appearance of bovine spongiform encephalopathy outside the United Kingdom (not a second wave resulting from codon 129 genotype differences). Two patients are excluded: 1 presymptomatic patient from the United States who received human growth hormone and died of an intercurrent illness and 1 dura mater recipient from the United Kingdom with disease onset in 1978.

The long incubation periods—years to decades—of these low-dose infections pose a particularly difficult problem for public health officials, whose recommendations may diminish the number of new cases but are impotent when it comes to preventing cases in already-infected persons in the preclinical phase of disease. It is worth remembering that the early recognition of iatrogenic sources of CJD was entirely because of a few remarkably astute neurologists, neurosurgeons, and, astonishingly, a pediatric endocrinologist who pursued the unlikely (and unpopular) diagnosis of CJD in a growth hormone recipient (*3*). It is true that some of these connections had the benefit of comparatively short intervals between the infecting events and the onset of CJD. It is especially fortunate from the standpoint of early recognition of the dura mater association that the interval of 19 months between the operation and onset of symptoms in the first case-patient was among the shortest on record for this form of iatrogenic CJD (Table 2).

**Table 2:** Incubation periods and clinical presentations of iatrogenic Creutzfeldt-Jakob disease, according to source of infection*

Source of Infection	No. cases	Mean incubation period, y (range)	Clinical signs†
Dura mater graft	228	12 (1.3–30)	Cerebellar, visual, dementia
Neurosurgical instruments	4	1.4 (1–2.3)	Visual, dementia, cerebellar
Stereotactic EEG needles	2	1.3, 1.7	Dementia, cerebellar
Corneal transplant	2	1.5, 27	Dementia, cerebellar
Growth hormone	226	17 (5–42)‡	Cerebellar
Gonadotropin	4	13.5 (12–16)	Cerebellar
Packed red blood cells§	3	6.5, 7.8, 8.3	Psychiatric, sensory, dementia, cerebellar

*EEG, electroencephalogram. †In order of decreasing frequency. ‡Averages and ranges were 13 (5–24) y in France; 20 (7–39) y in the United Kingdom; and 22 (10–42) y in the United States. §An additional asymptomatic but infected red-cell recipient died of an unrelated illness; another asymptomatic infected hemophilia patient who had been exposed to potentially contaminated factor VIII also died of an unrelated illness (neither is included in the table).

## Human Growth Hormone

The current worldwide total of growth hormone–associated cases of CJD is 226. Most cases occurred in France (119 cases/1,880 recipients; attack rate 6.3%), the United Kingdom (65 cases/1,800 recipients; attack rate 3.6%), and the United States (29 cases/7,700 recipients; attack rate 0.4%).

In France, further epidemiologic observations have revealed that all 119 cases occurred within a 1,170-patient cohort receiving treatment during a 20-month period, from December 1983 through July 1985, when there seems to have been substantial contamination resulting from sourcing and processing deficiencies. According to these numbers, the attack rate for the at-risk cohort in France increases to 10.2%. No new case has been identified since 2008. In the United Kingdom, no cohort pattern is evident, and cases continue to occur at an average rate of about 2 per year (only 1 in 2011). In the United States, CJD has not occurred in any patient who started treatment after 1977, when a highly selective column chromatography step was introduced into the purification protocol. Since 2003, only 2 new cases have been identified (1 in 2007 and 1 in 2009). An estimated ≈2,700 patients received treatment before 1977, so the attack rate in the United States for this at-risk cohort increases to 1.1% (*4*). The revised attack rates therefore become 10.2% in France, 3.6% in the United Kingdom, and 1.1% in the United States.

The methionine (M)/valine polymorphism at codon 129 of the *PRNP* gene has been examined in populations with and without CJD in many countries; results have varied (Table 3). Overall, it is clear that the M allele bestows substantial susceptibility to the sporadic and the iatrogenic forms of CJD; in consequence, the proportion of persons with MM homozygous genotype is overrepresented in both categories of disease (the sole exception occurred in UK growth hormone recipients, which led to speculation that a different strain of the pathogenic agent might have been disseminated) (*10*). It is also clear that, as a group, persons with heterozygous genotype had longer incubation periods than did those with homozygous genotype, particularly in France. Notwithstanding this statistical conclusion, it is noteworthy that several persons with MM homozygous genotype had incubation periods >30 years, including a patient with recently diagnosed CJD, whose incubation period was 42 years, the current world record for any type of iatrogenic disease.

**Table 3:** Comparison of *PRNP* codon 129 genotype frequencies and incubation periods in growth hormone– and dura mater–associated cases of iatrogenic CJD*

Category	MM	VV	Homozygotes	Heterozygotes
Population				
Healthy Caucasian, %†	40	10	50	50
European, with sporadic CJD, %	67	17	84	16
Healthy Japanese, %	92	0	92	8
Japanese, with sporadic CJD, (%)	97	1	98	2
Infection source				
Growth hormone				
France (111)				
Genotype frequency, %	54	15	69	31
Incubation period, y	12	9	11	17
United Kingdom (28)				
Genotype frequency, %	4	50	54	46
Incubation period, y	21	18	20	23
United States (11)				
Genotype frequency, %	55	18	73	27
Incubation period, y	21	18	20	23
Combined total (150)				
Genotype frequency, %	45	22	67	33
Incubation period, y	13	12	13	17
Dura mater				
Japan (54)‡				
Genotype frequency, %	96	0	96	4
Incubation period, y	16	NA	16	13
Countries other than Japan (54)§				
Genotype frequency, %	65	15	80	20
Incubation period, y	12	12	12	16
Combined total (108)				
Genotype frequency, %	81	7	88	12
Incubation period, y	14	12	14	16

*CJD, Creutzfeldt-Jakob disease; M, methionine; V, valine; NA, not applicable. All values are rounded to the nearest whole number. †Based on several large-scale population studies (*5*–*9*). ‡Personal communication from M. Yamama, Department of Neurology, Kanazawa University Hospital, Kanazawa, Japan. §Cases from France (11), Spain (11), Germany (10), Italy (8), the Netherlands (5), and 1 or 2 cases from each of 6 other countries with Caucasian populations.

Incubation periods for the total case population (not just those examined for the codon 129 genotype) ranged from 5 to 42 years (mean 17 years), based on the interval between the midpoint date of what was almost always a multiyear period of treatment and the onset of CJD symptoms; the actual date of infection is impossible to determine. Mean incubation periods for cases in the United States and New Zealand (patients received hormone made in the United States) were 22 and 26 years; United Kingdom, 20 years; and France, 13 years. The shorter incubation periods in France could have resulted partly from the narrower limit for the date of infection in France and are in accord with the mean incubation period of 13.5 years in the 4 gonadotropin recipients from Australia, for whom there is an even more precise date of infection. However, a greater contribution probably came from different infectious doses received by patients in the different countries. Among all patients, the clinical features were distinctive in that, unlike sporadic CJD, signs and symptoms almost never included dementia, which, if it occurred at all, was typically a late component of the clinical course.

## Dura Mater

The worldwide tally of dura mater–associated cases is 228, and new cases still continue to occur here and there, the most recent being individual cases in Austria, South Korea, and the Netherlands in 2011. If the pharmaceutical industry (in contrast to government-sponsored laboratories) comes away from the growth hormone story with an almost untainted record—only 1 case has been attributed to industrially prepared hormone (*11*)—the same cannot be said about the private sector producing dura mater grafts. The source of almost all infections was a manufacturer in Germany, B. Braun Melsungen AG, which has a worldwide distribution network, and the incidence of CJD appears to have more or less paralleled the frequency with which this source of dura mater was used. In Japan, it is estimated that as many as 20,000 patches may have been used each year, and the 142 cases in that country constitute two thirds of the global total. Nevertheless, the overall attack rate in the at-risk patient population in Japan is <0.03%. For the entire (worldwide) group of dura mater–recipient patients, incubation periods ranged from 1.3 to 30 years (mean 12 years), and, except in Japan, the clinical and neuropathologic features were similar to those of sporadic CJD. In Japan, approximately one third of the cases had atypical features (slow progression, noncharacteristic electroencephalogram tracings, plaque deposition, and an atypical prion protein molecular signature on Western blots), which suggested the possibility of 2 different strains of infecting agent (*12**,**13*). One patient had florid plaques and a pulvinar sign on magnetic resonance imaging, mimicking vCJD (*5*).

Evaluation of the influence of the codon 129 genotype is complicated by the fact that the population in Japan, among whom most cases occurred, has a high frequency of the M allele (>90%), which dominated sporadic and dura mater–associated forms of CJD (Table 3) (*6**–**9**,**14**,**15*). Among the cases in persons not from Japan, the distribution of genotypes approximated that found among patients with sporadic CJD, and, as with growth hormone–associated cases, incubation periods were somewhat longer for persons with heterozygous than with homozygous genotypes.

## Current Prevention Strategies

The best way to abolish secondary iatrogenic infections is, obviously, to prevent primary infections, but without a test to identify infected but asymptomatic persons, we cannot entirely eliminate the risk inherent in human-to-human tissue transfer. We are therefore obliged to rely on the default strategies of 1) identification and donor deferral of persons at higher than normal risk for CJD development and 2) inclusion of prion-reduction steps in the sterilization of penetrating instruments and the processing of therapeutic tissues and fluids.

Delineation of high-risk categories initially focused on precisely those groups of persons who were exposed to the known sources of iatrogenic disease: recipients of cadaveric dura mater grafts or pituitary-derived hormones. When vCJD started to occur, restrictions were also placed on donor time of residence in the most heavily infected regions—the United Kingdom and, to a lesser extent, continental Europe—and embargoes were placed on the importation of biological products from these regions. These deferral and import restrictions remain in place today and need some thoughtful reevaluation in view of the near extinction of all such sources of iatrogenic CJD. In the United States, there have been only 4 cases of dura mater–associated disease (the most recent in 2005) and no case of growth hormone–associated CJD for anyone who began treatment after 1977.

On the other hand, the possibility of iatrogenic infection resulting from transfer of tissues or fluids from persons who have contracted a prion disease from animals has not disappeared with the abating epidemics of bovine spongiform encephalopathy and vCJD. A few persons who may be experiencing a long incubation phase of vCJD still pose an obvious danger in the United Kingdom, but an underappreciated potential danger lies in 2 other animal diseases: scrapie and chronic wasting disease (CWD). Although scrapie-infected sheep tissues have been consumed for long enough (hundreds of years) to be considered harmless for humans, the same cannot be said about the atypical strains of scrapie that are beginning to displace the typical strains and with which we do not yet have enough experience to evaluate human pathogenicity. Similarly, we cannot declare with certainty that CWD poses no threat to humans, and CWD is continuing its unchecked spread across the United States and Canada with no guarantee that it will not become globally distributed in the years to come. One hunter has already put a group of unwitting persons at risk for infection by donating a deer, later found to have CWD, for consumption at a rural banquet in New York State (*16*); more such exposures are likely to occur as CWD continues its geographic expansion.

## Future Prevention Strategies

The issue of reducing risk by taking steps to inactivate prions is always a work in progress as new therapeutic products come into production and new methods to inactivate prions are discovered. The tried-and-true laboratory method of prion sterilization (1-hour exposures to either undiluted bleach or 1 N sodium hydroxide followed by steam autoclaving at 3 atmospheres pressure for 20 minutes) is applicable only to nonfragile instruments and not at all to living tissues. The surprising resistance of dura mater to 0.1 N sodium hydroxide (*17*) and of growth hormone to 6 M urea (*18*) led to their incorporation into processing protocols before being replaced by nondural tissue or synthetic patches and recombinant hormone. To reduce infectivity, blood, blood products, and other fluids can be subjected to nanofiltration and prion-affinity ligands (*19**–**22*), which should also be applicable to other biological products, for example, vaccine and stem cell cultures, should they be susceptible to infection (*23*). Fragile instruments such as endoscopes and electrodes remain a challenge, but new and gentler methods— alkaline cleaning solutions, phenolics, and gaseous hydrogen peroxide—have proven harmless to instruments and give a high, if not always complete, degree of prion inactivation (*24**–**26*).

The ongoing refinement of a quaking-induced conversion detection of the misfolded prion protein holds the best prospect of evolving into a sensitive and practical tool, but it has yet to be validated in blind testing of plasma from symptomatic patients or in presymptomatic persons, even more rigorous but necessary (*27**,**28*). It may be necessary to use scrapie-infected animals for presymptomatic validation because only 1 group of humans could furnish appropriate samples—asymptomatic carriers of CJD-inducing mutations—and putting together and testing a reasonable number of such samples will take years to accomplish.

The total numbers of cases for the 2 major causes of iatrogenic CJD during the past 40 years (226 growth hormone cases and 228 dura mater cases) are amazingly close and are likely to remain so after the few additional long-incubating cases finally surface in the next few years. The combination of appropriate blood donor deferrals and the incorporation of tissue, fluid, and instrument infectivity–reduction steps should continue to hold the sources of potential iatrogenic disease to a minimum until such time as a practical screening test for inapparent infection is validated for human use.

## References

[1] Duffy P, Wolf J, Collins G, DeVoe AB, Streeten B, Cowen D. Letter: possible person-to-person transmission of Creutzfeldt-Jakob disease. N Engl J Med. 1974;290:692–3. 10.1056/NEJM1974032129012204591849

[2] Brown P, Brandel J-P, Preece M, Sato T. Iatrogenic Creutzfeldt-Jakob disease: the waning of an era. Neurology. 2006;67:389–93. 10.1212/01.wnl.0000231528.65069.3f16855204

[3] Brown P. Human growth hormone therapy and Creutzfeldt-Jakob disease: a drama in three acts. Pediatrics. 1988;81:85–92.3275936

[4] Abrams JY, Schonberger LB, Belay ED, Maddox RA, Leschek EW, Mills JL, Lower risk of Creutzfeldt-Jakob disease in pituitary growth hormone recipients initiating treatment after 1977. J Clin Endocrinol Metab. 2011;96:E1666–9. 10.1210/jc.2011-135721816775

[5] Wakisaka Y, Santa N, Doh-ura K, Kitamoto T, Ibayashi S, Iida M, Increased asymmetric pulvinar magnetic resonance imaging signals in Creutzfeldt-Jakob disease with florid plaques following a cadaveric dura mater graft. Neuropathology. 2006;26:82–8. 10.1111/j.1440-1789.2006.00638.x16521484

[6] Soldevila M, Calafell F, Andrès AM, Yagüe J, Helgason A, Stefánsson K, Prion susceptibility and protective alleles exhibit marked geographic differences. Hum Mutat. 2003;22:104–5. 10.1002/humu.915712815603

[7] Nurmi MH, Bishop M, Strain L, Brett F, McGuigan C, Hutchison M, The normal population distribution of *PRNP* codon 129 polymorphism. Acta Neurol Scand. 2003;108:374–8. 10.1034/j.1600-0404.2003.00199.x14616310

[8] Mercier G, Diéterlen F, Lucotte G. Population distribution of the methionine allele at the *PRNP* codon 129 polymorphism in Europe and the Middle East. Hum Biol. 2008;80:181–90. 10.3378/1534-6617(2008)80[181:PDOTMA]2.0.CO;218720902

[9] Doh-ura K, Kitamoto T, Sakaki Y, Taateishi J. CJD discrepancy. Nature. 1991;353:801–2. 10.1038/353801b01682813

[10] Brandel J-P, Preece M, Brown P, Croes E, Laplanche J-L, Agid Y, Distribution of codon 129 genotype in human growth hormone–treated CJD patients in France and the UK. Lancet. 2003;362:128–30. 10.1016/S0140-6736(03)13867-612867116

[11] Furtner M, Gelpi E, Kiechl S, Knoflach M, Zangerl A, Gotwald T, Iatrogenic Creutzfeldt-Jakob disease 22 years after human growth hormone therapy: clinical and radiological features. J Neurol Neurosurg Psychiatry. 2008;79:229–31. 10.1136/jnnp.2007.12226718202218

[12] Noguchi-Shinohara M, Hamaguchi T, Kitamoto T, Sato T, Nakamura Y, Mizusawa H, Clinical features and diagnosis of dura mater graft–associated Creutzfeldt-Jakob disease. Neurology. 2007;69:360–7. 10.1212/01.wnl.0000266624.63387.4a17646628

[13] Yamada M, Noguchi-Shinohara M, Hamaguchi T, Nozaki I, Kitamoto T, Sato T, Dura mater graft–associated Creutzfeldt-Jakob disease in Japan: clinicopathological and molecular characterization of the two distinct subtypes. Neuropathology. 2009;29:609–18. 10.1111/j.1440-1789.2008.00987.x19659940

[14] Nozaki I, Hamaguchi T, Sanjo N, Noguchi-Shinohara M, Sakai K, Nakamura Y, Prospective 10-year surveillance of human prion diseases in Japan. Brain. 2010;133:3043–57. 10.1093/brain/awq21620855418

[15] Ladogana A, Puopolo M, Croes EA, Budka H, Jarius C, Collins S, Mortality from Creutzfeldt-Jakob disease and related disorders in Europe, Australia, and Canada. Neurology. 2005;64:1586–91. 10.1212/01.WNL.0000160117.56690.B215883321

[16] Garruto RM, Reiber C, Alfonso MP, Gastrich H, Needham K, Sunderman S, Risk behaviors in a rural community with a known point-source exposure to chronic wasting disease. Environ Health. 2008;7:31. 10.1186/1476-069X-7-3118577220PMC2453121

[17] Diringer H, Braig HR. Infectivity of unconventional viruses in dura mater. Lancet. 1989;1:439–40. 10.1016/S0140-6736(89)90035-42563815

[18] Pocchiari M, Peano S, Conz A, Eshkol A, Maillard F, Brown P, Combination ultrafiltration and 6 M urea treatment of human growth hormone effectively minimizes risk from potential Creutzfeldt-Jakob disease virus contamination. Horm Res. 1991;35:161–6. 10.1159/0001818941806470

[19] Yunoki M, Tanaka H, Urayama T, Hattori S, Ohtani M, Ohkubo Y, Prion removal by nanofiltraion under different experimental conditions. Biologicals. 2008;36:27–36. 10.1016/j.biologicals.2007.04.00517890100

[20] Cardone F, Simoneau S, Arzel A, Puopolo M, Berardi VA, Abdel-Haq H, Comparison of nanofiltration efficacy in reducing infectivity of centrifuged versus ultracentrifuged 263K scrapie-infected brain homogenates in “spiked” albumin solutions. Transfusion. 2011. Epub ahead of print. 10.1111/j.1537-2995.2011.03425.x22082124

[21] Gregori L, Gurgel PV, Lathrop JT, Edwardson P, Lambert BC, Carbonell RG, Reduction in infectivity of endogenous transmissible spongiform encephalopathies present in blood by adsorption to selective affinity resins. Lancet. 2006;368:2226–30. 10.1016/S0140-6736(06)69897-817189034

[22] Heger A, Bailey A, Neisser-Svae A, Ertl M, Römisch J, Svae TE. Removal of prion infectivity by affinity ligand chromatography during OctaplasLG manufacturing—results from animal bioassay studies. Vox Sang. 2011. Epub ahead of print. 10.1111/j.1423-0410.2011.01563.x22070802

[23] Piccardo P, Cervenakova L, Vasilyeva I, Yakovleva O, Bacik I, Cervenak J, Candidate cell substrates, vaccine production, and transmissible spongiform encephalopathies. Emerg Infect Dis. 2011;17:2262–9. 10.3201/eid1712.11060722172513PMC3311205

[24] Fichet G, Comoy E, Duval C, Antioga K, Dehen C, Charbonnier A, Novel methods for disinfection of prion-contaminated medical devices. Lancet. 2004;364:521–6. 10.1016/S0140-6736(04)16810-415302195

[25] Fichet G, Antioga K, Comoy E, Deslys JP, McDonnell G. Prion inactivation using a new gaseous hydrogen peroxide sterilization process. J Hosp Infect. 2007;67:278–86. 10.1016/j.jhin.2007.08.02017942185

[26] Fichet G, Harrison J, McDonnell G. Reduction of risk of prion transmission on surgical devices with effective cleaning processes. Zentr Steril. 2007;15:418–37.

[27] Orrú CD, Wilham JM, Raymond LD, Kuhn F, Schroeder B, Raeber AJ, Prion disease blood test using immunoprecipitation and improved quaking-induced conversion. MBiol. 2011;3:e00078-11 [cited 2012 Mar 31]. http://mbio.asm.org/content/2/3/e00078-11.full10.1128/mBio.00078-11PMC310178221558432

[28] Orrú CD, Wilham JM, Vascellari S, Hughson AG, Caughey B. New generation QuIC assays for prion seeding activity. Prion. 2012;6. Epub ahead of print.10.4161/pri.19430PMC708209122421206

# Review article: Iatrogenic Creutzfeldt-Jakob Disease, Final Assessment

**DOI:** 10.3201/eid1806.120116

**Authors:** Paul Brown, Jean-Philippe Brandel, Takeshi Sato, Yosikazu Nakamura, Jan MacKenzie, Robert G. Will, Anna Ladogana, Maurizio Pocchiari, Ellen W. Leschek, Lawrence B. Schonberger

**Affiliations:** Centre à l’Energie Atomique, Fontenay-aux-Roses, France (P. Brown);; Institut National de la Santé et de la Recherche Médicale, Paris, France (J.-P. Brandel);; Nanohana Clinic, Tokyo, Japan (T. Sato);; Jichi Medical University, Yakushiji, Japan (Y. Nakamura);; Western General Hospital, Edinburgh, Scotland, UK (J. MacKenzie, R.G. Will);; Istituto Superiore de Sanità, Rome, Italy (A. Ladogana, M. Pocchiari);; National Institutes of Health, Bethesda, Maryland, USA (E.W. Leschek);; Centers for Disease Control and Prevention, Atlanta, Georgia, USA (L.B. Schonberger)

## Medscape CME Quiz

## Earning CME Credit

To obtain credit, you should first read the journal article. After reading the article, you should be able to answer the following, related, multiple-choice questions. To complete the questions (with a minimum 70% passing score) and earn continuing medical education (CME) credit, please go to www.medscape.org/journal/eid. Credit cannot be obtained for tests completed on paper, although you may use the worksheet below to keep a record of your answers. You must be a registered user on Medscape.org. If you are not registered on Medscape.org, please click on the New Users: Free Registration link on the left hand side of the website to register. Only one answer is correct for each question. Once you successfully answer all post-test questions you will be able to view and/or print your certificate. For questions regarding the content of this activity, contact the accredited provider, CME@medscape.net. For technical assistance, contact CME@webmd.net. American Medical Association’s Physician’s Recognition Award (AMA PRA) credits are accepted in the US as evidence of participation in CME activities. For further information on this award, please refer to http://www.ama-assn.org/ama/pub/category/2922.html. The AMA has determined that physicians not licensed in the US who participate in this CME activity are eligible for *AMA PRA Category 1 Credits*™. Through agreements that the AMA has made with agencies in some countries, AMA PRA credit may be acceptable as evidence of participation in CME activities. If you are not licensed in the US, please complete the questions online, print the certificate and present it to your national medical association for review.

## Article Title: Iatrogenic Creutzfeldt-Jakob Disease, Final Assessment

## CME Questions

**1. Your patient is a 50-year-old woman with a complicated medical history. She has required treatment with multiple biologic agents and had surgery with placement of a cadaveric ligament in her knee.**

**She read an article regarding prion disease and is concerned regarding her risk for illness. According to the current review, what have been the principal sources of iatrogenic Creutzfeldt-Jakob disease (CJD)?**

A. Corneal transplants and packed red blood cells

B. Instruments and gonadotropins

C. Dura mater grafts and growth hormone

D. Packed red blood cells and platelets

**2. Most cases of iatrogenic CJD caused by human growth hormone are reported in which of the following countries?**

A. Ghana, Ivory Coast, and Nigeria

B. Vietnam, Cambodia, and Laos

C. France, the United Kingdom, and the United States

D. Brazil, Colombia, and Peru

**3. What can you tell this patient about characteristics of iatrogenic CJD?**

A. Homozygotes for the methionine valine polymorphism had longer incubation times

B. The mean incubation period is approximately 2 years

C. Dementia was the most common early manifestation of CJD

D. One manufacturer accounted for nearly all infections associated with dura mater grafts

**4. What are the most prominent new threats for CJD?**

A. Scrapie and chronic wasting disease

B. The consumption of “bush meat”

C. Coinfection with coxsackievirus

D. Wider consumption of meat by children

## Activity Evaluation

**Table:** 

1. The activity supported the learning objectives.				
Strongly Disagree				Strongly Agree
1	2	3	4	5
2. The material was organized clearly for learning to occur.				
Strongly Disagree				Strongly Agree
1	2	3	4	5
3. The content learned from this activity will impact my practice.				
Strongly Disagree				Strongly Agree
1	2	3	4	5
4. The activity was presented objectively and free of commercial bias.				
Strongly Disagree				Strongly Agree
1	2	3	4	5
